# Improving bi-exponential fitting of autofluorescence lifetime decay data through whole-cell binning

**DOI:** 10.1117/1.BIOS.3.4.045001

**Published:** 2026-11-11

**Authors:** Olivia I. Kolenc, Divya M. Gollapalli, Alan E. Woessner, Jocelyn Martinez, Alex J. Walsh, Kyle P. Quinn

**Affiliations:** a University of Arkansas, Department of Biomedical Engineering, Fayetteville, Arkansas, United States; b Tufts University, Department of Biomedical Engineering, Medford, Massachusetts, United States; c Texas A&M University, Department of Biomedical Engineering, College Station, Texas, United States

**Keywords:** multiphoton microscopy, fluorescence lifetime imaging, autofluorescence, metabolic imaging, NADH

## Abstract

**Significance:**

Fluorescence lifetime imaging (FLIM) analysis commonly involves multi-exponential fitting of fluorescence decays, which requires relatively high signal-to-noise ratios (SNRs). However, autofluorescence presents a weaker signal compared with exogenous fluorophores, which can make fitting challenging. Becker & Hickl’s SPCImage (SPCI) is commonly used for bi-exponential fitting of NAD(P)H autofluorescence decays, but its accuracy has not been robustly evaluated.

**Aim:**

We evaluate the accuracy of bi-exponential modeling of NAD(P)H FLIM data from live cells and provide an alternative fitting algorithm that allows for photon binning of whole cells for low SNR applications.

**Approach:**

Simulated fluorescence lifetime decay data and NADH solutions were used to test the accuracy of fitting in both SPCI and a custom-written MATLAB algorithm. FLIM images collected from live cells using varying integration times were also evaluated.

**Results:**

Results show consistently higher error for SPCI in fluorescence decay parameters compared with the custom-written MATLAB algorithm. Whole-cell binning greatly improved the accuracy of all lifetime decay parameters, allowing for shorter integration times during acquisition.

**Conclusions:**

Our custom-written MATLAB algorithm can provide a flexible alternative fitting tool that allows for photon binning of whole cells and substantially more accurate parameter estimation.

Statement of Discovery This work utilizes a unique spatial binning strategy for fluorescence lifetime analysis of NAD(P)H that greatly improves the accuracy of extracted fit parameters compared with commercial software. This will enable faster acquisition from live cells and improved estimations of the protein-binding status of metabolic cofactors.

## 1 Introduction

Multiphoton microscopy techniques are well suited to measure cell metabolism noninvasively in three-dimensional (3D) tissues without exogenous stains or dyes.^1^ Two-photon excited fluorescence (TPEF) can be elicited from many naturally occurring fluorophores (autofluorescence) through the simultaneous absorption of two photons of half the energy, resulting in photon emission in the ultraviolet and visible light range.^2^^,^^3^ TPEF in combination with fluorescence lifetime imaging (FLIM) can measure how long a fluorophore remains excited before returning to its ground state post-excitation.^4^^,^^5^ This measurement can vary for individual fluorophores based on their microenvironment and other molecular interactions, rendering it a valuable tool for metabolic characterization. In particular, the autofluorescence of two metabolic cofactors, the reduced form of nicotinamide adenine dinucleotide (NADH), the phosphorylated form of NADH (NADPH), and flavin adenine dinucleotide (FAD), has been quantified to assess metabolic changes in a variety of cells and tissues, including skin, brain, muscle, and liver.^6^^–^^10^

NADH and NADPH have nearly identical emission spectra and are often collectively referred to as NAD(P)H.^3^^,^^11^ NAD(P)H and FAD can be spatially and temporally resolved using time-correlated single photon counting (TCSPC) FLIM, which is acquired through the repeated measure of the time between excitation and the detection of emitted photons.^12^ This technique is useful in noninvasively assessing cellular metabolism both *in vitro* and *in vivo* based on the protein binding state of NADH, NADPH, and FAD, which can be distinguished from the fluorescence lifetimes associated with their free-floating and protein-bound states.^13^^–^^15^ NAD(P)H free and bound lifetimes are reported to average around 0.4 ns and range from 1.9 to 5.7 ns, respectively.^12^^,^^16^^–^^18^ Bound FAD has a shorter lifetime, ranging from 0.003 to 4.55 ns, whereas free FAD is reported to average around 2.6 ns.^5^^,^^9^^,^^19^ The lifetimes of these two molecules, as well as their quantum yield (a measure of the efficiency of fluorescence emission relative to photon absorption), vary depending on their conformation and the specific cellular protein they are bound to.^17^^,^^20^^,^^21^

However, investigating cellular metabolism using autofluorescence signals poses several challenges, particularly with the inherently poor quantum yields of endogenous fluorophores compared with exogenous fluorophores. In comparison to extrinsic fluorophores, NAD(P)H and FAD have quantum yields (0.02 and 0.033, respectively) an order of magnitude lower than common fluorescent dyes such as fluorescein (0.86) and rhodamine B (0.59).^22^^–^^24^ Due to their low quantum yield, collecting sufficient photons for NAD(P)H and FAD FLIM analysis can be a challenge, particularly with *in vitro* imaging, which typically presents lower signal-to-noise ratios (SNRs).

FLIM collection often requires integration times of at least 2 min to obtain an adequate SNR for resolving multiexponential decays, which involves total photon counts in the range of several thousands to tens of thousands of photons per pixel.^25^ Multiple FLIM analysis methods are available, with fitting to a bi-exponential decay model the most common method for NADH data, although fit-free phasor analysis and emerging methods employing statistical denoising techniques, such as PCA, and machine and deep learning are gaining prominence.^26^^–^^30^ Bi-exponential fitting is highly reliant on the photon count recorded per pixel and requires a well-sampled fluorescence decay curve to produce an accurate fit of the decay kinetics. When using bi-exponential fitting, a time-of-arrival histogram is constructed from the totality of the photons collected per pixel and then fit to a bi-exponential model to produce a fluorescence decay curve at each pixel, given by

I(t)=I0(α1e−tτ1+α2e−tτ1). (1)

Fluorescence lifetime parameters corresponding to the short and long lifetime components ($\tau_{1}$, $\tau_{2}$), and their respective relative contributions ($\alpha_{1}$, $\alpha_{2}$), can then be calculated from the decays.^31^ Typically, $\tau_{1}$, $\tau_{2}$, $\tau_{M}$, and $\alpha_{2}\%$ values are reported, where $\tau_{M}$ represents the average lifetime given by

$$\tau_{M}=(\alpha_{1}*\tau_{1})+(\alpha_{2}*\tau_{2}).$$ (2)

Besides the assumption of the number of fluorophore species present, bi-exponential fitting of fluorescence decays typically employs an instrument response function (IRF) to account for the temporal response of the imaging system.^32^^,^^33^ Shift and offset terms are also often incorporated to account for variations in the temporal alignment of the fluorescence decay and as a baseline correction for instrumental noise.^31^^,^^34^ Poor fitting accuracy for FLIM data has often been attributed to low photon counts and low SNR, fluorescence lifetimes shorter than the IRF, as well as poor initial parameter estimates applied within fitting algorithms. However, there has been a lack of in-depth examinations of accuracy in these analysis methods. There is a need for refined analysis methods to accurately decipher metabolic data from autofluorescent signals.

A variety of tool options exist for performing bi-exponential fits of FLIM data, including software such as PicoQuant, and a number of packages based in MATLAB, Python, R, and ImageJ.^29^^,^^30^^,^^35^^–^^38^ Many laboratories with microscopes equipped with TCSPC FLIM modules use Becker & Hickl’s SPCImage (SPCI) software because of its inclusion with the hardware.^39^^–^^41^ SPCI is employed in a wide range of applications, including the evaluation of fluorescent dyes and single molecules, analyzing Förster resonance energy transfer (FRET) sensors for the measurement of protein interactions and conformations for cell biology, biosensing, and cell and tissue imaging. SPCI is often used for the analysis of extrinsic fluorescence but has been increasingly implemented in autofluorescence-based metabolic imaging. However, SPCI has not been well evaluated for its accuracy in analyzing autofluorescence decays.

In this study, we evaluated the accuracy of bi-exponential decay curve fitting in SPCI by simulating NAD(P)H autofluorescence decay curves. An alternative approach to enable bi-exponential fitting using a custom-written MATLAB algorithm was also developed and used to fit the same simulated NAD(P)H decays. The bi-exponential fitting results of the simulated NAD(P)H curves were compared between this custom algorithm and SPCI. The values resulting from bi-exponential fitting were also verified using an NAD(P)H solution to ensure the simulated NAD(P)H decay curves match literature and experimental values. Finally, as a means to boost SNR, we introduce a precision binning approach for cell images that significantly reduces FLIM integration time during data collection.

## 2 Methods

### 2.1 Simulation of Single-Pixel NAD(P)H Fluorescence Lifetime Decays

The bi-exponential fluorescence decay model given in Eq. (1)^25^^,^^31^^,^^32^ was used to construct 128 fluorescence decay curves in MATLAB based on the typical reported parameters for NAD(P)H lifetimes: $\tau_{1}(0.3-0.4\;\mathrm{ns})$, $\tau_{2}(1.9-5.7\;\mathrm{ns})$, $\alpha_{1}$ (70% to 80%), and $\alpha_{2}$ (15% to 30%).^5^^,^^9^^,^^12^^,^^16^^–^^19^ An experimentally derived IRF was collected from urea crystals on a custom-built inverted multiphoton microscope (Bruker, Nikon, Shinagawa, Japan) equipped with a Ti:Sapphire laser (MaiTai, SectraPhysics, Milpitas, California, United States) and convolved with the ideal decays produced by random combinations of NAD(P)H lifetime parameters. Decays reflect four levels of SNR, ranging from high SNR (100,000 photons), moderate SNR (10,000), moderate-low SNR (5000 photons), and low SNR (1000 photons). The four levels of total photon counts in each decay capture the range in signal quality observed in NADH FLIM imaging, from the most ideal to severely limited SNR. Poisson noise was applied to all the decays using the built-in MATLAB function, poissrnd.m. Decay curves were written to 128×128  pixel SPCI files (.sdt).

### 2.2 SPCI Bi-exponential Fitting of Single-Pixel Simulated NADH Decays

The Becker & Hickl’s SPCImage 8.3 was used to evaluate the SPCI files containing the simulated single-pixel decay curves, where an incomplete multi-exponential model with two components, zero binning, and the same IRF used to create the simulated decay curves. A percent error calculation defined by

$$\%\text{Error}=|(\frac{\text{Simulated}-\text{True}}{\text{True}})|*100$$ (3)

was computed to evaluate SPCI’s baseline accuracy.

### 2.3 Custom MATLAB Fitting Algorithm

MATLAB R2020b was used to construct an iterative curve fitting algorithm utilizing MATLAB’s native nonlinear least-squares solver, lsqcurvefit.m, utilizing the interior-point algorithm. Our function creates a fluorescence decay fit based on iterative estimations of FLIM parameters. A scaling factor (sf) and FLIM parameter estimations were used to create an ideal decay, which can be described by

Iideal=sf*((α1e(−tτ1)+α2e(−tτ2))). (4)

This ideal decay is then convolved with an IRF shifted in time to match the response of the collected experimental data and adjusted with an offset term to account for potential differences in photon counts by shifting the decay in the y direction so that the peaks of the IRF and the decay align. This operation can be described by

$$I_{\text{fit}}=(I_{\text{ideal}}⊗{\mathrm{IRF}}_{\text{shifted}})+\text{offset}.$$ (5)

To ensure the most accurate fit of the decay data, the “best fit” of the decay data was selected from 10 iterations by the lowest residual sum of squares between the decay data and the estimated decay fit generated by the function. The same percent error calculation was made as with the results of SPCI fitting to evaluate the custom algorithm’s accuracy.

### 2.4 Validation of Fluorescence Lifetimes Obtained from MATLAB Algorithm Fitting

An NADH and lactate dehydrogenase (LDH) solution was created to independently test the curve fitting results obtained from the MATLAB algorithm. First, the concentration of lactate dehydrogenase (LDH) needed to generate an LDH-NADH solution with a free fraction, $\alpha_{1}$, of 0.6, was calculated from the equations, $[\text{Enzyme}]=\frac{\left[\mathrm{NADH}](1-\alpha_{1}\right)}{F*S}$ and $F=\frac{\alpha_{1}*[\mathrm{NADH}]}{\alpha_{1}*[\mathrm{NADH}]+K_{d}}$.^42^ These equations were used to develop and validate a computational model and have been further validated experimentally in previous work.^43^ The expression for the fraction of occupied binding sites, F, was substituted into the enzyme concentration equation to yield a single equation that relates the free fraction $\alpha_{1}$, the concentration of NADH [NADH], the concentration of the enzyme [Enzyme], the dissociation constant $K_{d}$, and the number of binding sites S. To minimize self-quenching, an NADH concentration of 50  μM was used. LDH has four binding sites per enzyme molecule (S=4).^44^ A $K_{d}$ value of 4.2  μM was used for the dissociation constant of NADH to LDH at room temperature, 20°C.^44^ Solving the equations provided an LDH enzyme concentration of 5.7  μM.

To create the LDH-NADH solution, a 50 mM NADH stock (1000×) was first generated by dissolving 354.9 mg of NADH disodium salt (Sigma) into 10 mL of 1× phosphate-buffered saline (Gibco). This stock was subsequently diluted 1:10 in PBS to obtain a 5 mM NADH working stock (100×). The final solution, containing 50  μM NADH and 5.7  μM LDH, was prepared by mixing 10  μL of the 100× NADH stock with 0.801 mL of a 7.12  μM LDH solution (Sigma) and 189  μL of 1× PBS, followed by gentle vortexing. For imaging, 200  μL of either the 100× NADH stock or the 50  μM LDH-NADH solution was transferred into separate 35 mm glass-bottom imaging dishes.

Fluorescence lifetime imaging of the LDH-NADH solution was performed using a custom-built inverted multiphoton fluorescence microscope (Marianas, 3i) equipped with a 40× water-immersion objective (NA 1.1), providing a field of view of ∼270×270  μm. NADH was excited at 750 nm using a titanium:sapphire femtosecond laser (Chameleon, COHERENT), with excitation power maintained between 20 and 25 mW. Fluorescence lifetime data were acquired using TCSPC electronics (SPC-150N, Becker &Hickl). Images were collected of 256×256  pixels with a pixel dwell time of 50  μs and five-frame integration, resulting in a total acquisition time of ∼60  s per image.

Captured fluorescence lifetime images of the LDH-NADH solution were analyzed using SPCI and the MATLAB fitting algorithm to determine their accuracies. Using MATLAB, all the photons within an image were summed together to produce a single decay curve and then fit using the custom MATLAB algorithm described using the IRF of the system. Within SPCI, an incomplete multi-exponential model with two components, maximum binning of 10, and the same IRF from the system was applied.

### 2.5 Simulated NAD(P)H FLIM Cell Images for Whole-Cell Precision Binning

Simulated NAD(P)H FLIM cell images were created to test the accuracy of FLIM analysis of a cell image in SPCI. A total of 18 simulated NAD(P)H FLIM cell images were produced from a binary cell mask manually traced from a real cell image. Cells occupied an average of 38,347±14,694.5  pixels within the mask. To simulate FLIM data with low SNR, a previously generated simulated NAD(P)H FLIM decay curve with a photon count of 1000 was randomly selected and assigned to each pixel within cells in each image. Random Poisson noise was added to all pixels in each image using the built-in MATLAB function, poissrnd.m. These simulated NAD(P)H FLIM cell images were analyzed in SPCI after applying an incomplete multi-exponential model with two components, a maximum binning of 10 (a 21×21  pixel neighborhood). Using a whole-cell binning approach in MATLAB, photons in each individual cell were combined within the cell masks to produce a single decay curve for each cell and then fit using the custom MATLAB fitting algorithm.

### 2.6 Precision Binning Validation in NIH 3T3 Cells

NIH 3T3 mouse embryonic fibroblast cells (ATCC) were cultured using minimal essential medium (MEM, Gibco, Grand Island, New York, United States), supplemented with 10% fetal bovine serum (FBS, Gibco) in the absence of antibiotics. Cells were passaged onto 35 mm, poly-D-lysine coated glass bottom dishes (MatTek) at 40% to 50% confluence for multiphoton imaging. A total of six dishes, with three random fields of view captured per dish, were imaged within a microincubator (Warner Instruments, Holliston, Massachusetts, United States) maintained at 37°C with 5% ${\mathrm{CO}}_{2}$. FLIM was captured using a custom-built inverted multiphoton microscope (Bruker, Nikon) equipped with a Ti:Sapphire laser (SpectraPhysics) via a 60× water immersion objective (1.2 NA). Images were captured at a resolution of 1024×1024  pixels (181.5  μm×181.5  μm; pixel size 0.177  μm) using 12 mW of power at the objective and a dwell time of 0.4  μs at integration times of 30, 60, and 120 s for each field. Each image field was collected at all three integration times. NAD(P)H autofluorescence was isolated at 755 nm excitation using a GaAsP detector with a 460 nm (±20  nm) emission filter. The same analysis approach using whole-cell precision binning described for the simulated cell images was applied to evaluate 18 individual cells, where each cell had a corresponding 30, 60, and 120 s integration time. Each cell was manually traced using the image corresponding to the 120 s integration time to produce a binary mask containing a single cell and applied to all three integration times. Cells occupied an average of 39,530±15,955  pixels within the masks.

### 2.7 Statistical Analysis

Statistical analysis was performed in JMP Pro 18.0.2. For each assessment, a linear mixed effects model was used to compare the method used: SPCI or the custom MATLAB algorithm. The method of fitting was treated as a fixed effect, with an additional fixed factor corresponding to the variable of interest (photons, cell, or integration time) and their interaction (method × photons, method × cell, or method × integration time). A random effect (either individual simulation or cell identifiers) was included to account for paired comparisons for the same simulation or cell. A post-hoc Tukey’s honest significance test was used to identify significant differences. An additional linear effects model comparing methods by cell with cell identifiers as a random effect was applied to the simulated image data to obtain cell by cell differences. All error bars were constructed using one standard deviation from the mean. The confidence interval bands in Figs. 1, 3, and 4 were estimated using the built-in MATLAB function nlpredci.m.

**Fig. 1:**
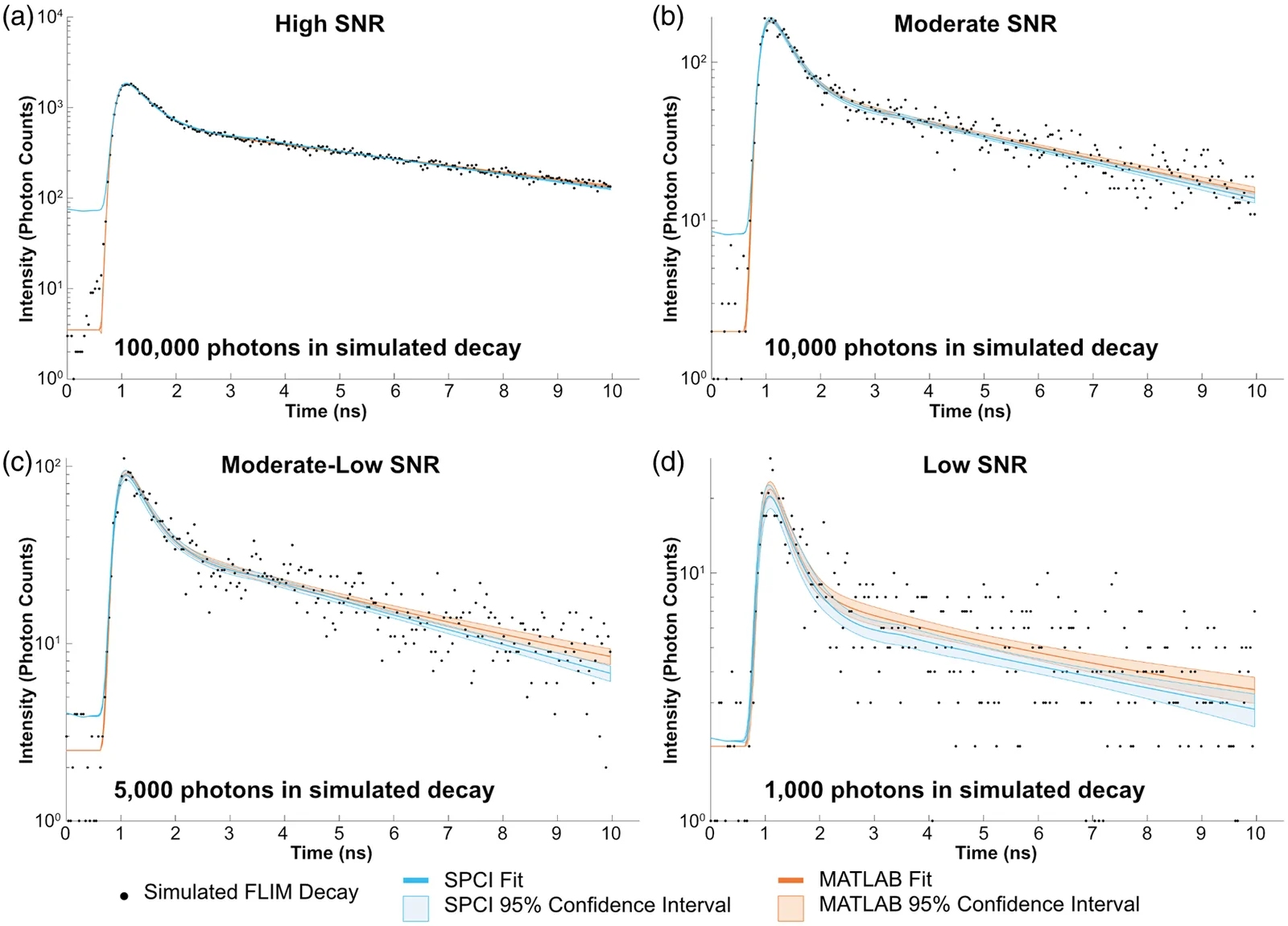
Comparison between bi-exponential fits of a representative simulated NADH single pixel decay curve with (a)–(d) decreasing SNR using a custom MATLAB fitting algorithm (solid orange) and SPCI (solid blue). A 95% confidence band is shown for both MATLAB (shaded orange) and SPCI (shaded blue).

## 3 Results

### 3.1 Impact of Noise on Bi-exponential Fitting Accuracy in SPCI and a Custom-Written MATLAB Algorithm

In comparing the results from SPCI and the developed MATLAB algorithm, the observed trend revealed visually diverging fits as photon counts decreased (Fig. 1). This range of photon counts included high SNR data (100,000 photon counts) often from extrinsic fluorophores, moderate (10,000 photon counts), and moderate-low (5000 photon counts) commonly used for NADH analysis, as well as low SNR data (1000 photon counts) often obtained from cell cultures without spatial binning. At a photon count of 100,000 (high SNR) and 10,000 (moderate SNR), both MATLAB and SPCI yielded comparable fits with minimal deviation [Figs. 1(a)–1(b)]. As SNR decreased to moderate-low and low levels [Figs. 1(c)–1(d)], MATLAB demonstrated a better ability to fit the visual center of the decay curve, displaying more resilience to noisy outliers compared with SPCI.

To quantify the baseline accuracy of SPCI compared with MATLAB, the percent error for each of the FLIM parameters ($\tau_{1}$, $\tau_{2}$, $\tau_{M}$, $\alpha_{1}\%$, and $\alpha_{2}\%$) was calculated based on the known simulated values and the results of fitting with each method. The percent error for both SPCI and MATLAB trended similarly, increasing as photon counts and SNR decreased (Fig. 2). However, SPCI was frequently less accurate for many parameter estimates in comparison to MATLAB, exhibiting higher percent error particularly with lower photon counts (Fig. 2). For $\tau_{1}$, MATLAB was significantly more accurate than SPCI for decays with high (p=0.0018), moderate (p<0.0001), and moderate-low (p<0.0001) SNR but was not significantly different (p=0.1143) for decays with low SNR [Fig. 2(a)]. For $\tau_{2}$, SPCI showed a substantial increase in percent error with decreasing SNR, with the percent error significantly higher compared with MATLAB for all SNR levels except for the highest (1000 to 10,000 photon counts: p<0.0001; 100,000 photon counts: p=1.0) [Fig. 2(b)]. In the case of $\tau_{M}$, MATLAB was significantly more accurate for decays with moderate and low SNR (10,000 and 1000 photon counts: p<0.0001), but significantly less accurate for moderate-low SNR (5000 photon counts: p=0.0331) (Fig. 2(e)). When averaged across all SNR levels, MATLAB demonstrated a percent error of 12.4%, 7.94%, and 9.13% for $\tau_{1}$, $\tau_{2}$, and $\tau_{M}$, respectively, whereas SPCI exhibited an average percent error of 18.64%, 21.18%, and 15.17%, respectively. For $\alpha_{1}\%$ and $\alpha_{2}\%$, MATLAB also produced a significantly lower percent error than SPCI for decays with moderate-low SNR ($\alpha_{1}\%$: p=0.0334 and $\alpha_{2}\%$: p=0.0203) and low SNR ($\alpha_{1}\%$ and $\alpha_{2}\%$: p<0.0001) [Figs. 2(c)–2(d)], with no significant difference for decays with high SNR ($\alpha_{1}\%$: p=0.6252 and $\alpha_{2}\%$: p=0.5494) and moderate SNR ($\alpha_{1}\%$: p=0.9995 and $\alpha_{2}\%$: p=0.9996). At a photon count of 1000, the lowest SNR decays present a greater challenge in accuracy as they represent SNR much lower than what is typically considered acceptable. Across all noise levels for $\alpha_{1}\%$ and $\alpha_{2}\%$, MATLAB had an average percent error of 3.15% and 9.64%, respectively, whereas SPCI had an average percent error of 4.34% and 13.27%, respectively.

**Fig. 2:**
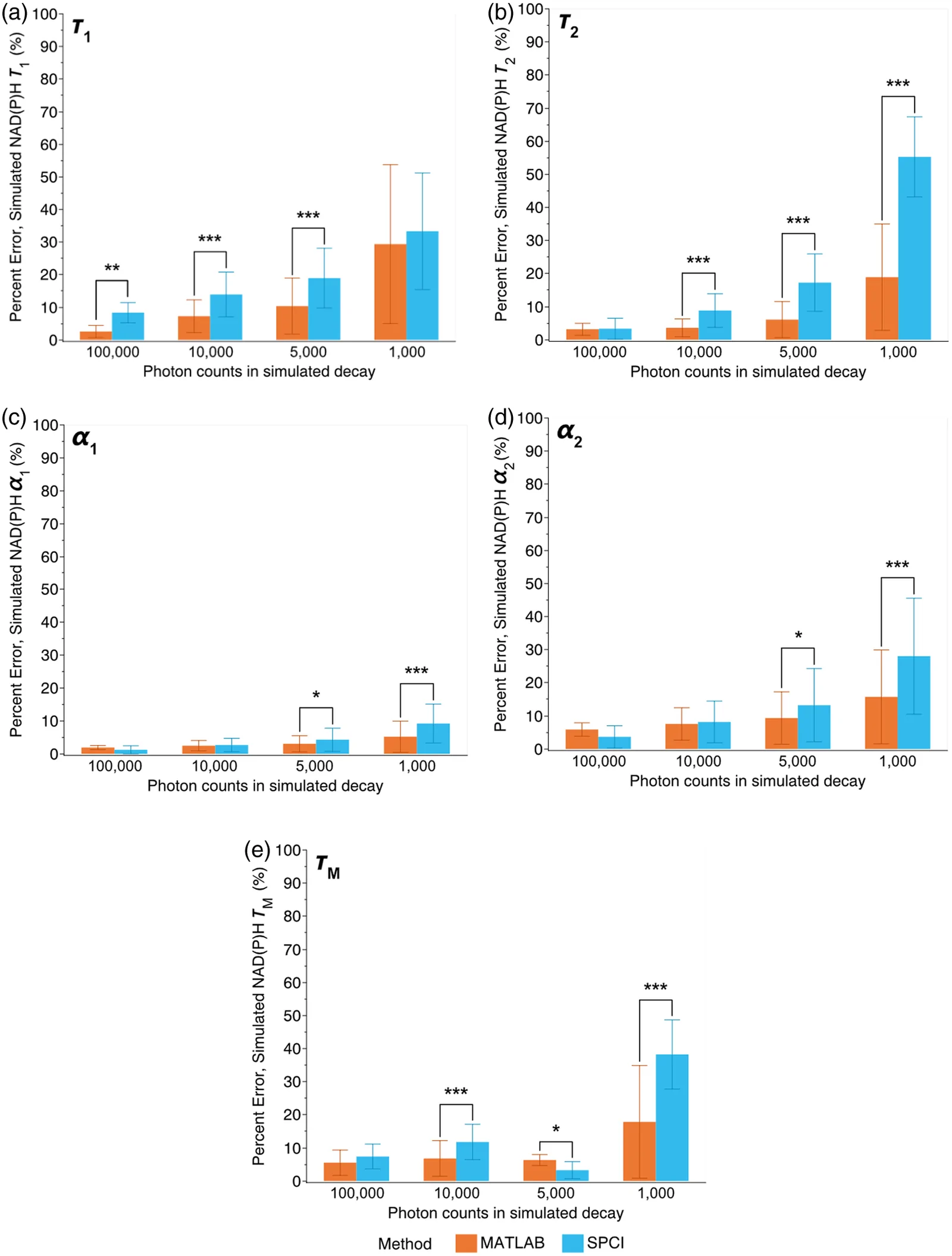
Percent error for FLIM parameters obtained from bi-exponential fitting of simulated NADH single pixel decay curves with decreasing SNR using a custom MATLAB fitting algorithm and SPCI. As photon counts and SNR decrease, the error in (a) short/free lifetime component ($\tau_{1}$), (b) long/bound lifetime component ($\tau_{2}$), (c) free lifetime contribution ($\alpha_{1}$), (d) bound lifetime contribution ($\alpha_{2}$), and (e) average lifetime ($\tau_{M}$) increases (* denotes p≤0.05, *** denotes p≤0.001).

### 3.2 Experimental Validation of Lifetime Parameter Estimation

To validate the accuracy of the custom-written MATLAB algorithm in estimating lifetime parameters, a known solution of 50  μM NADH and 5.7  μM lactate dehydrogenase (LDH) with a predefined $\alpha_{1}\%$ contribution of 60% was used. LDH, known for its role in catalyzing the conversion of lactate to pyruvate, engages in the reduction of ${\mathrm{NAD}}^{+}$ to NADH in both the cellular cytoplasm and mitochondrial matrix. FLIM measurements of the solution were acquired, and the decays fitted using both MATLAB and SPCI. Employing a whole-image binning approach in MATLAB where all photons within the field of view were summed resulted in a notably less noisy decay curve compared with SPCI with binning of 2 or 10 applied (Fig. 3). The FLIM parameters obtained with MATLAB were $\tau_{1}=0.266\;\mathrm{ns}$, $\tau_{2}=1.366\;\mathrm{ns}$, $\tau_{M}=0.671\;\mathrm{ns}$, and $\alpha_{1}\%=62.1\%$. In comparison, SPCI with a bin of 2 produced FLIM parameters of $\tau_{1}=0.320\;\mathrm{ns}$, $\tau_{2}=1.008\;\mathrm{ns}$, $\tau_{M}=0.457\;\mathrm{ns}$, and $\alpha_{1}\%=48.6\%$. In addition, SPCI with a bin of 10 yielded FLIM parameters of $\tau_{1}=0.32\;\mathrm{ns}$, $\tau_{2}=1.315\;\mathrm{ns}$, $\tau_{M}=0.676\;\mathrm{ns}$, and $\alpha_{1}\%=64.2\%$.

### 3.3 Comparative Evaluation of a Whole-Cell Binning Approach for Low SNR FLIM

A whole-cell binning approach for *in vitro* cell cultures was tested to determine whether a sufficient SNR improvement could be achieved by increasing photon counts by pooling pixels beyond the limits of SPCI, as demonstrated with the whole-image binning approach for LDH-NADH solutions (Fig. 3). Our precision whole cell binning approach sums photons within the boundaries of a cell to produce a lifetime decay curve with higher photon counts, and thus, improved SNR for each cell in an image (Fig. 4). This approach improves upon the constraints of SPCI, where the maximum pixel neighborhood that may be used for photon binning is limited to 21×21  pixels. To test this binning method, a simulated cell image was generated with Poisson noise across all pixels and the lowest photon count examined to mimic a low SNR environment [Fig. 4(a)]. Maximum binning in SPCI for decay curves with lower SNR does not adequately improve SNR for a sufficiently accurate fit of the decay [Fig. 4(b)] based on our simulated decay analysis (Figs. 1 and 2).

**Fig. 3:**
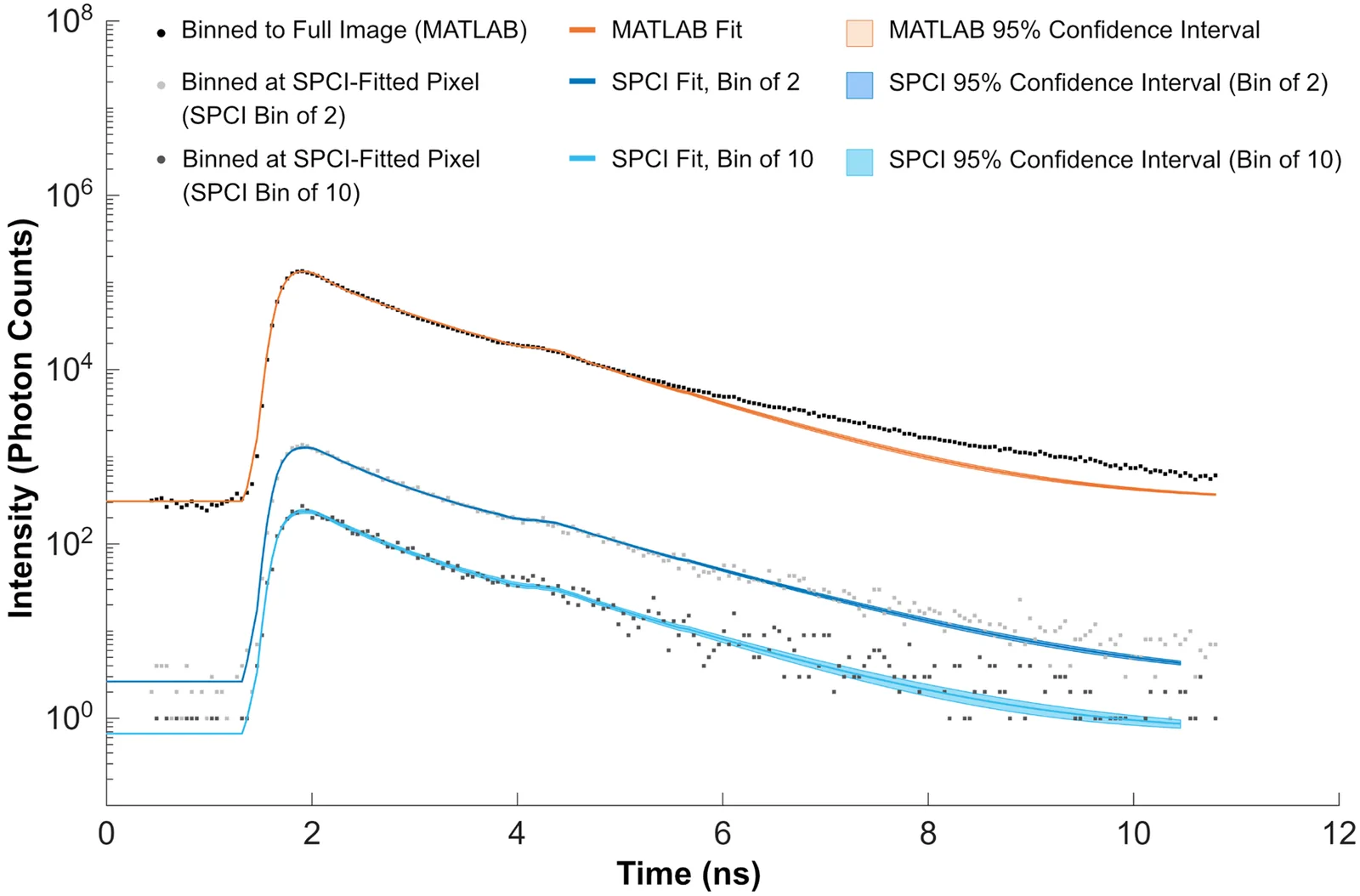
Comparison between bi-exponential fits of a FLIM decay for a LDH-NADH solution using a custom MATLAB fitting algorithm and SPCI. A whole-image binning approach was used in MATLAB. For SPCI, a binning of 10 and a binning of 2 were used. A 95% confidence band is shown for both MATLAB (shaded orange) and SPCI (shaded blue).

**Fig. 4:**
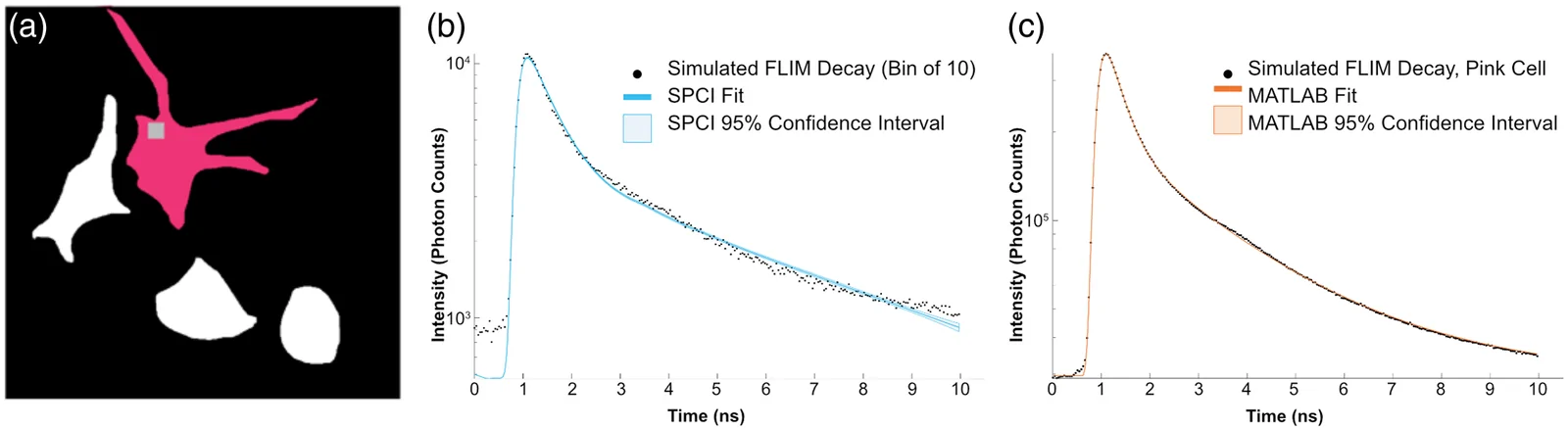
Comparison of simulated NADH fluorescence decays after using (b) the maximum binning of 10 in SPCI and (c) whole-cell binning in MATLAB. (a) A mask from a manually traced cell image was used to simulate an NADH FLIM image of cells. The gray square in the pink highlighted cell (a) denotes the 21×21  pixel neighborhood corresponding to maximum binning in SPCI. Representative decays from the pink highlighted cell (b) and (c) illustrate the difference in decay noise resulting from the application of whole-cell binning in MATLAB, which (c) eliminates noise from the decay, in contrast to (b) SPCI’s maximum binning. A 95% confidence band is shown for both MATLAB (shaded orange) and SPCI (shaded blue).

The comparison between whole-cell binning and fitting with MATLAB and traditional SPCI fitting revealed greater predictive accuracy using MATLAB for all FLIM parameters (p<0.0001) (Fig. 5). SPCI exhibited higher percent errors across each parameter and had average percent errors of 35.77% for $\tau_{1}$, 141.27% for $\tau_{2}$, 73.19% for $\tau_{M}$, 4.92% for $\alpha_{1}\%$, and 16.2% for $\alpha_{2}\%$. By contrast, our MATLAB algorithm produced average percent errors of 0.61% for $\tau_{1}$, 1.66% for $\tau_{2}$, 6.12% for $\tau_{M}$, 2.00% for $\alpha_{1}\%$, and 6.86% for $\alpha_{2}\%$. Comparisons between cells further emphasized these differences, with significantly lower percent error for each cell in the simulated image compared with SPCI with maximum binning for all parameters (cell 1: $\tau_{1}$, $\tau_{2}$, $\tau_{M}\;1$, $\alpha_{1}\%$ and $\alpha_{2}\%$ p<0.0001; cell 2: $\tau_{1}$, $\tau_{2}$, $\tau_{M}\;1$, $\alpha_{2}\%$ p<0.0001, and $\alpha_{1}\%$ p=0.0041; cell 3: $\tau_{1}$, $\tau_{2}$, $\tau_{M}1$, $\alpha_{2}\%$ p<0.0001, and $\alpha_{1}\%$ p=0.0006; cell 4: $\tau_{1}$, $\tau_{2}$, $\tau_{M}\;1$, $\alpha_{2}\%$ p<0.0001, and $\alpha_{1}\%$ p=0.0005) (Fig. 5).

**Fig. 5:**
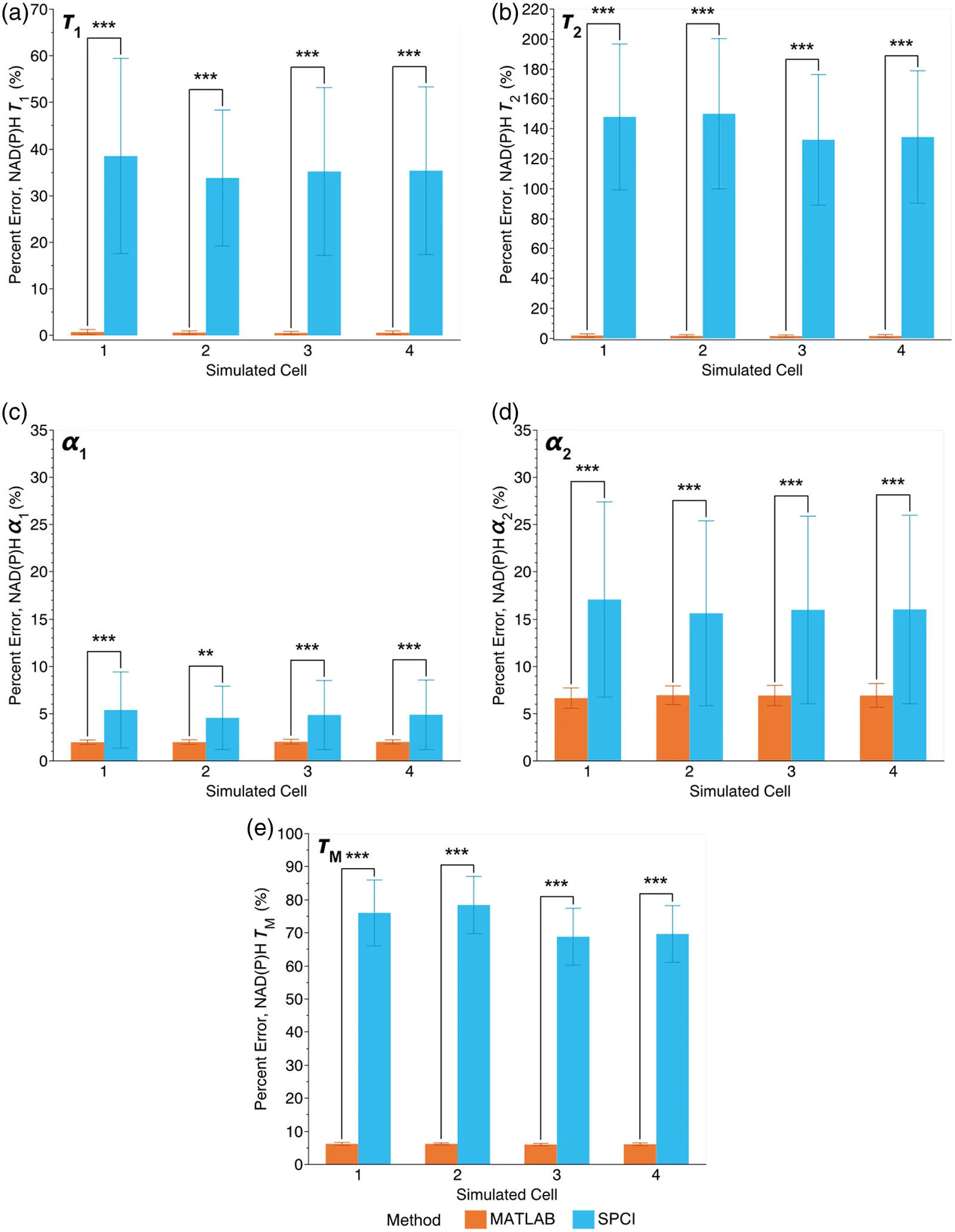
Percent error for FLIM parameters obtained from bi-exponential fitting of simulated NADH FLIM cell images using a custom MATLAB fitting algorithm using a whole-cell binning approach (orange) and SPCI using the maximum binning of 10 (blue). For all lifetime parameters, including (a) short/free lifetime component ($\tau_{1}$), (b) long/bound lifetime component ($\tau_{2}$), (c) free lifetime contribution ($\alpha_{1}$), (d) bound lifetime contribution ($\alpha_{2}$), and (e) average lifetime ($\tau_{M}$), error was significantly lower using a whole-cell binning approach (* denotes p≤0.05, ** denotes p≤0.01, *** denotes p≤0.001).

### 3.4 Impact of a Whole-Cell Binning Approach on FLIM Integration Time

The efficacy of the precision whole-cell binning approach in reducing the integration time required for reliable FLIM decay data analysis was investigated using varying FLIM integration times with NIH 3T3 cells at low power. As integration time increased from 30 to 120 s, cell FLIM images exhibited a noticeable improvement in SNR, with greater numbers of photons within each cell mask [Fig. 6(a)]. SPCI responded inconsistently across integration times, whereas MATLAB remained consistent across integration times for each FLIM parameter. Overall, the responses for $\tau_{2}$ and $\tau_{M}$ were most dependent on the fitting method. Integration time was a significant factor for $\tau_{1}$ (p=0.019), $\tau_{2}$ (p<0.0001), and $\tau_{M}$ (p<0.0001), whereas an overall significant difference between MATLAB and SPCI alone was found only for $\tau_{M}$ (p<0.0001). Importantly, all FLIM parameters across all three integration times utilizing the whole-cell binning approach in MATLAB were not significantly different from each other (30 s: $\tau_{1}$ p=0.1203, $\tau_{2}$ p=0.159, $\tau_{M}$ p=0.9996, $\alpha_{1}\%$ and $\alpha_{2}\%$ p=0.9832; 60 s: $\tau_{1}$ p=0.1718, $\tau_{2}$ p=0.1413, $\tau_{M}$ p=0.9998, $\alpha_{1}\%$ and $\alpha_{2}\%$ p=0.9714; 120 s: $\tau_{1}$ p=0.09, $\tau_{2}$ p=0.1723, $\tau_{M}$ p=0.9865, $\alpha_{1}\%$ and $\alpha_{2}\%$ p=0.9896). However, significant differences at the 30-s integration time emerged between SPCI and MATLAB across all integration times for $\tau_{2}$ (p<0.0003), and $\tau_{M}$ (p<0.0002) [Fig. 6(b) and 6(e)].

**Fig. 6:**
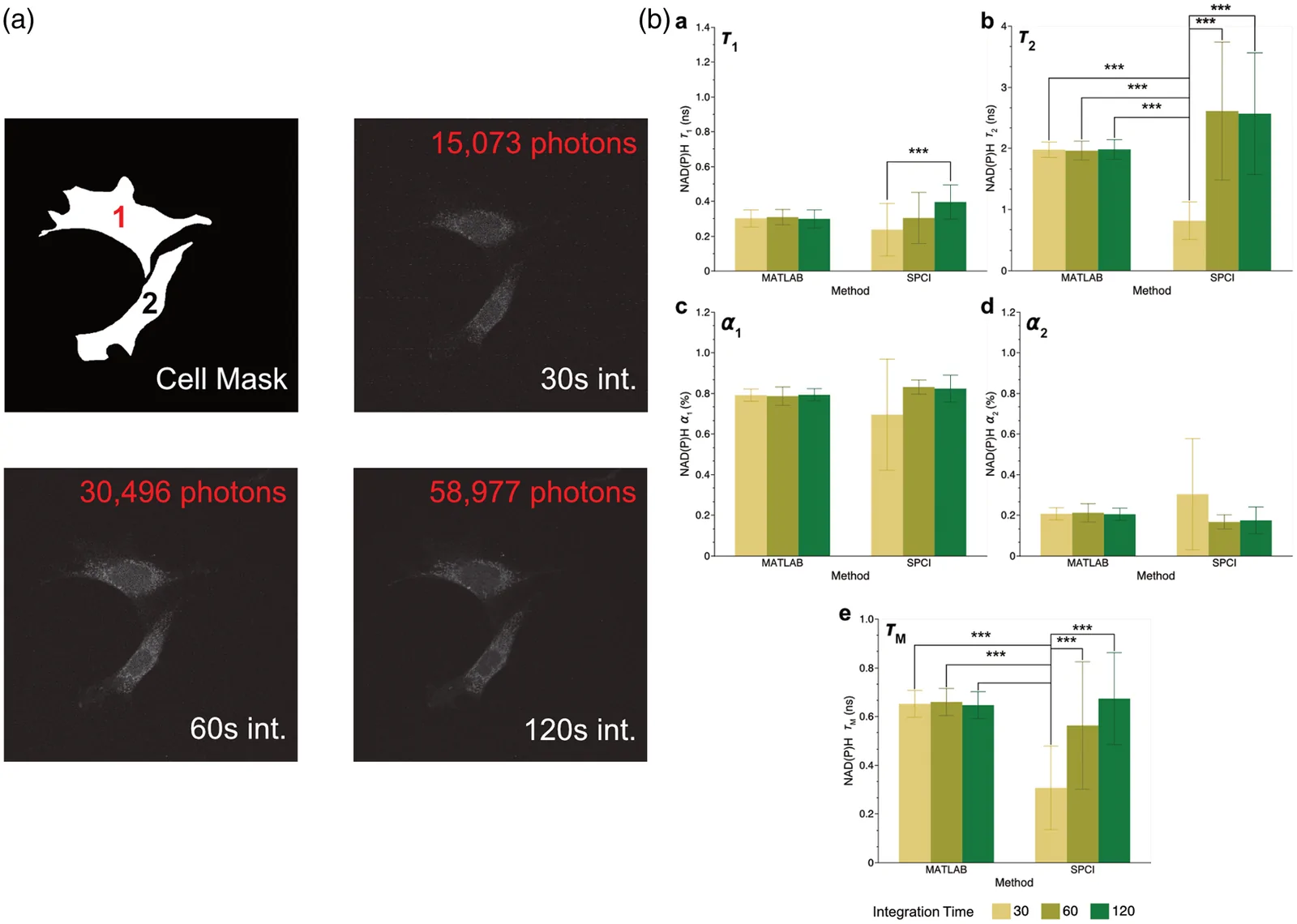
Application of whole-cell binning using a MATLAB fitting algorithm to real cell NAD(P)H FLIM. (a) A representative set of NAD(P)H FLIM intensity images of NIH 3T3 cells at 30 s (yellow), 60 s (olive), and 120 s (green) FLIM integration times, including the number of photons collected for cell 1 at each integration time, and their corresponding mask. (b) Comparison of the FLIM parameters obtained from the MATLAB whole-cell binning approach and SPCI analysis with maximum binning. ($\tau_{1}$, short/free lifetime component; $\tau_{2}$, long/bound lifetime component; $\tau_{M}$, average lifetime; $\alpha_{1}$, free lifetime contribution; * denotes p≤0.05, ** denotes p≤0.01, *** denotes p≤0.001).

## 4 Discussion

Bi-exponential fitting of FLIM decay data is highly impacted by the low SNR inherent to measuring autofluorescence and significantly influences the accuracy of FLIM-based metabolic measurements. Particularly, in the context of metabolic measurements through NAD(P)H FLIM, the use of SPCI for bi-exponential fitting of autofluorescence decay data has become more widespread in recent years. However, the suitability of standard FLIM fitting software for such analyses has remained largely unexplored until now.

Our study offers a comprehensive assessment of SPCI’s accuracy in handling bi-exponential fitting of low SNR FLIM data characteristic of autofluorescence-based measures of metabolism and introduces a custom MATLAB algorithm for more precise and accurate fitting, which can leverage flexible photon binning of whole cells to mitigate low SNR, further improve accuracy, and reduce FLIM integration times. Overall, SPCI was found to be limited in its ability to mitigate the effects of noise during fitting of poor SNR data and displayed higher error for the majority of FLIM parameters in comparison to the MATLAB algorithm developed in this study. The fitting accuracy of the MATLAB algorithm was further improved over SPCI through binning the photons of all pixels within a user-defined mask without the inclusion of background pixels, making the MATLAB algorithm better suited for analyzing low SNR autofluorescence data that typically is averaged on a cell-by-cell basis after fitting.

Simulated NAD(P)H single pixel decay curves provided a baseline understanding of the error SPCI is subject to across high, moderate, moderate-low, and low SNR NAD(P)H FLIM data. The photon count range paired with Poisson noise simulated the effect of various sources of noise, such as instrument noise, ambient light, or photon counting errors. Variation of FLIM parameters associated with NAD(P)H presented a wide range of potential real-world combinations for a more robust evaluation of the limits of bi-exponential fitting within SPCI and the custom-written MATLAB fitting algorithm. Estimation of FLIM parameters in SPCI and MATLAB using a predefined LDH-NADH solution experimentally validated the simulated estimation of FLIM parameters in MATLAB and produced similar results in both MATLAB and SPCI. Literature values for the lifetime of NADH bound to LDH are mixed, where LDH-NADH solutions range anywhere from 0.26 to 4 ns.^21^^,^^42^^,^^45^ Variations in the preparations of enzymatic mixtures with different solvents, NADH:enzyme ratios, and differences in FLIM system setups can account for the variability in the reported values.

Our findings suggest that SPCI has a limited capability in managing noise-related challenges during bi-exponential fitting. When no binning was applied to either fitting approach, SPCI had predominantly higher error for all FLIM parameters as SNR decreased with decreased photon counts in comparison to the custom-written MATLAB algorithm developed for this study. For simulated single decay curves, $\tau_{1}$, $\tau_{2}$, and $\tau_{M}$ exhibited the highest percent error for SPCI. The percent error for $\tau_{M}$ was not as high as that of $\tau_{2}$, which may be attributed to the higher accuracy of $\tau_{1}$ and $\alpha_{1}\%$, because these parameters are included in the average lifetime calculation. The improved accuracy observed with the MATLAB-based algorithm for many of the parameters likely stems from differences in how the nonlinear optimization is initialized and constrained. Commercial software like SPCI is designed to process entire images rapidly, which requires internal optimizations and convergence thresholds that balance speed with accuracy. By contrast, our MATLAB algorithm relies on a transparent model allowing for strict control over fitting parameters and tolerances. This level of control can allow users to prioritize accuracy over speed, which is likely helpful when dealing with low-SNR NADH autofluorescence decays. SPCI can mitigate SNR issues and reduce some fitting error by increasing the SNR through spatial binning of neighboring pixels. However, SPCI is limited to a maximum binning of 10, which corresponds to a pixel neighborhood of 21×21  pixels. This neighborhood essentially functions as a kernel, averaging photons in the neighborhood around each pixel across the entire image. This method of binning can incorporate a large number of pixels that should not be included in analysis in images where cells or tissues have many edges bordered by background pixels, other undesired fluorophores, or tissue regions outside of regions of interest. By taking a whole-cell binning approach in MATLAB, a larger pixel neighborhood can be selected for binning. This approach effectively mitigates low SNR issues by pooling more photons in a specific region of interest, thereby amplifying the signal. Importantly, this approach eliminates the inclusion of unwanted background pixels in lifetime calculations, improving the accuracy of estimating FLIM parameters from a bi-exponential fit, thus facilitating more accurate cell-to-cell, or region-to-region, comparisons. It is important to note that improvement in SNR utilizing this approach relies on selected regions of interest larger than a 21×21  pixel neighborhood.

This precision whole-cell binning approach is not limited to *in vitro* analysis and is applicable to any maskable region of interest for which autofluorescence data were collected. The maskable region also is not required to be manually traced, as performed in this study, and may be isolated utilizing automated methods. For example, in *in vivo* studies of skin autofluorescence, thin regions of interest corresponding to the epithelial layer must be isolated to characterize cell metabolism through NAD(P)H FLIM.^6^^,^^46^ Our custom MATLAB fitting algorithm together with the precision binning approach can easily be adapted to studies such as this to provide greater accuracy of key FLIM parameters and flexibility to summarize data over any region of interest. In addition, this approach would be beneficial for applications where acquisition speed and laser power at the objective are major concerns, such as in tracking metabolic fluctuations during embryo development.^47^^–^^49^ Faster acquisition times at lower laser powers induce fewer fluorophore excitation events, reducing the number of photons collected; this results in low SNR FLIM data that cannot be reliably analyzed. Furthermore, the custom MATLAB fitting algorithm and precision whole-cell binning approach could be expanded to account for variations in the bound lifetime of NAD(P)H due to interactions with different binding sites or binding to various enzymes.^13^^,^^15^^,^^50^ The precision whole-cell binning approach is well suited to advance explorations of multi-exponential fitting by addressing limitations with overfitting noisier FLIM decay curves^51^ due to its ability to mitigate the negative effects of poor SNR.

Although autofluorescence FLIM analysis was greatly improved by the application of the custom-written fitting algorithm together with the precision whole-cell binning approach, pooling photons in this way produces a single decay for each cell or region of interest instead of maintaining a high-resolution image with a FLIM decay per pixel, as with SPCI. Lifetime mapping of an image is no longer available, eliminating analysis of specific regions within a cell or region of interest, or visualization of lifetime parameters. However, most FLIM analysis is averaged across an image field, or individual cells or regions of interest are traced and averaged as a post-processing step. This results in reported fluorescent lifetimes as averages of each of the FLIM parameters. As this type of FLIM analysis does not take full advantage of the available spatial resolution, the outcome of the precision whole-cell binning approach provides a similar level of information as is habitually reported.

## 5 Conclusion

Our findings challenge the conventional use of SPCI for bi-exponential fitting in metabolic analysis and propose the MATLAB algorithm developed here as a more appropriate choice specifically for the analysis of low SNR FLIM data, especially when coupled with the precision whole-cell binning approach. SPCI offers a fast and accurate fitting solution for analyzing high SNR FLIM data typically collected with exogenous fluorophores, but our study highlights significant underperformance during NAD(P)H imaging with low SNR. This study not only addresses the limitations of SPCI but also opens a new avenue for expediting FLIM data acquisition without compromising accuracy. The potential of the precision whole-cell binning approach, as highlighted in this study, offers promising implications for enhancing the efficiency of cellular metabolism studies.

## Data Availability

The MATLAB code written for bi-exponential fitting of fluorescence decay data and precision-binning of whole cells is available on GitHub (https://github.com/kylepquinn). Other data underlying the results presented in this paper are not publicly available at this time but may be obtained from the authors upon reasonable request.

## References

[1] Kolenc O. I. Quinn K. P., “Evaluating cell metabolism through autofluorescence imaging of NAD(P)H and FAD,” Antioxid. Redox Signal. 30(6), 875–889 (2019). 10.1089/ars.2017.7451 PMC6352511 29268621

[2] Awasthi K. et al., “Sensitive detection of intracellular environment of normal and cancer cells by autofluorescence lifetime imaging,” J. Photochem. Photobiol. B 165, 256–265 (2016). 10.1016/j.jphotobiol.2016.10.023 27842280

[3] Galeotti T. et al., “On the fluorescence of NAD(P)H in whole-cell preparations of tumours and normal tissues,” Eur. J. Biochem. 17(3), 485–496 (1970). 10.1111/j.1432-1033.1970.tb01191.x 4395441

[4] Chen L.-C. et al., “The potential of label-free nonlinear optical molecular microscopy to non-invasively characterize the viability of engineered human tissue constructs,” Biomaterials 35(25), 6667–6676 (2014). BIMADU 0142-9612 10.1016/j.biomaterials.2014.04.080 PMC4106121 24854093

[5] Islam M. S. et al., “pH dependence of the fluorescence lifetime of FAD in solution and in cells,” Int. J. Mol. Sci. 14(1), 1952–1963 (2013).1422-0067 10.3390/ijms14011952 PMC3565358 23334475

[6] Jones J. D. et al., “Quantifying age-related changes in skin wound metabolism using in vivo multiphoton microscopy,” Adv. Wound Care 9(3), 90–102 (2020). 10.1089/wound.2019.1030 PMC6985773 31993251

[r7] Kunz W. S. Gellerich F. N., “Quantification of the content of fluorescent flavoproteins in mitochondria from liver, kidney cortex, skeletal muscle, and brain,” Biochem. Med. Metab. Biol. 50(1), 103–110 (1993). BMMBES 0885-4505 10.1006/bmmb.1993.1051 8373630

[r8] Thrane A. S. et al., “In vivo NADH fluorescence imaging indicates effect of aquaporin-4 deletion on oxygen microdistribution in cortical spreading depression,” J. Cereb. Blood Flow Metab. 33(7), 996–999 (2013). 10.1038/jcbfm.2013.63 PMC3705443 23611872

[r9] Kunz W. S., “Spectral properties of fluorescent flavoproteins of isolated rat liver mitochondria,” Febs Lett. 195(1-2), 92–96 (1986). FEBLAL 0014-5793 10.1016/0014-5793(86)80137-5 3753688

[10] Mazumder N. et al., “Fluorescence lifetime imaging of alterations to cellular metabolism by domain 2 of the hepatitis C virus core protein,” PLoS One 8(6), e66738 (2013). POLNCL 1932-6203 10.1371/journal.pone.0066738 PMC3691201 23826122

[11] Patterson G. H. et al., “Separation of the glucose-stimulated cytoplasmic mitochondrial NAD(P)H responses in pancreatic islet beta cells,” Proc. Natl. Acad. Sci. U. S. A. 97(10), 5203–5207 (2000). 10.1073/pnas.090098797 PMC25806 10792038

[12] Blacker T. S. Duchen M. R., “Investigating mitochondrial redox state using NADH and NADPH autofluorescence,” Free Rad. Biol. Med. 100, 53–65 (2016). FRBMEH 0891-5849 10.1016/j.freeradbiomed.2016.08.010 PMC5145803 27519271

[13] Blacker T. S. et al., “Separating NADH and NADPH fluorescence in live cells and tissues using FLIM,” Nat. Commun. 5(1), 3936 (2014). NCAOBW 2041-1723 10.1038/ncomms4936 PMC4046109 24874098

[r14] Lakowicz J. R. et al., “Fluorescence lifetime imaging of free and protein-bound NADH,” Proc. Nat. Acad. Sci. U. S. A. 89(4), 1271–1275 (1992). 10.1073/pnas.89.4.1271 PMC48431 1741380

[15] Skala M. C. et al., “In vivo multiphoton microscopy of NADH and FAD redox states, fluorescence lifetimes, and cellular morphology in precancerous epithelia,” Proc. Nat. Acad. Sci. U. S. A. 104(49), 19494–19499 (2007). 10.1073/pnas.0708425104 PMC2148317 18042710

[16] Berezin M. Y. Achilefu S., “Fluorescence lifetime measurements and biological imaging,” Chem. Rev. 110(5), 2641–2684 (2010). CHREAY 0009-2665 10.1021/cr900343z PMC2924670 20356094

[r17] Blinova K. et al., “Distribution of mitochondrial NADH fluorescence lifetimes: steady-state kinetics of matrix NADH interactions,” Biochemistry 44(7), 2585–2594 (2005). BICHAW 0006-2960 10.1021/bi0485124 15709771

[r18] Huang S. Heikal A. A. Webb W. W., “Two-photon fluorescence spectroscopy and microscopy of NAD(P)H and flavoprotein,” Biophys. J. 82(5), 2811–2825 (2002). BIOJAU 0006-3495 10.1016/S0006-3495(02)75621-X PMC1302068 11964266

[19] Islam S. D. M. et al., “Fluorescence quenching of flavin adenine dinucleotide in aqueous solution by pH dependent isomerisation and photo-induced electron transfer,” Chem. Phys. 295(2), 137–149 (2003). CMPHC2 0301-0104 10.1016/j.chemphys.2003.08.013

[20] Blacker T. S. Duchen M. R. Bain A. J., “NAD(P)H binding configurations revealed by time-resolved fluorescence and two-photon absorption,” Biophys. J. 122(7), 1240–1253 (2023). 10.1016/j.bpj.2023.02.014 PMC10111271 36793214

[21] Cannon T. M. et al., “Characterization of NADH fluorescence properties under one-photon excitation with respect to temperature, pH, and binding to lactate dehydrogenase,” OSA Contin. 4(5), 1610–1625 (2021). 10.1364/OSAC.423082 PMC8367293 34458690

[22] Xu C. Webb W. W., “Measurement of two-photon excitation cross sections of molecular fluorophores with data from 690 to 1050 nm,” J. Opt. Soc. Am. B 13(3), 481–491 (1996). 10.1364/JOSAB.13.000481

[r23] Valeur B. Brochon J., New Trends in Fluorescence Spectroscopy: Applications to Chemical and Life Sciences, Springer Science & Business Media (2012).

[24] Gorbunova I. A. et al., “Determination of fluorescence quantum yields and decay times of NADH and FAD in water–alcohol mixtures: the analysis of radiative and nonradiative relaxation pathways,” J. Photochem. Photobiol. A: Chem. 436, 114388 (2023). 10.1016/j.jphotochem.2022.114388

[25] Walsh A. J. et al., “Temporal binning of time-correlated single photon counting data improves exponential decay fits and imaging speed,” Biomed. Opt. Express 7(4), 1385–1399 (2016). BOEICL 2156-7085 10.1364/BOE.7.001385 PMC4929649 27446663

[26] Hu L. et al., “Comparison of phasor analysis and biexponential decay curve fitting of autofluorescence lifetime imaging data for machine learning prediction of cellular phenotypes,” Front. Bioinform. 3, 1210157 (2023). 10.3389/fbinf.2023.1210157 PMC10342207 37455808

[r27] Smith J. T. et al., “Fast fit-free analysis of fluorescence lifetime imaging via deep learning,” Proc. Natl. Acad. Sci. U. S. A. 116(48), 24019–24030 (2019). 10.1073/pnas.1912707116 PMC6883809 31719196

[r28] Heliot L. Leray A., “Simple phasor-based deep neural network for fluorescence lifetime imaging microscopy,” Sci. Rep. 11(1), 23858 (2021). 10.1038/s41598-021-03060-x PMC8668934 34903737

[r29] Marois A. L. Suhling K., “Quantitative live cell FLIM imaging in three dimensions,” in Multi-Parametric Live Cell Microscopy of 3D Tissue Models, pp. 31–48 (2017). 10.1007/978-3-319-67358-5_3 29080129

[30] Soltani S. et al., “Denoising of fluorescence lifetime imaging data via principal component analysis,” Sci. Rep. 15(1), 40258 (2025). SRWSDA 10.1038/s41598-025-24022-7 PMC12623853 41249821

[31] Becker W., The bh TCSPC Handbook, Becker & Hickl GmbH (2023).

[32] Fazel M. et al., “Fluorescence lifetime: beating the IRF and interpulse window,” Biophys. J. 122(4), 672–683 (2023). 10.1016/j.bpj.2023.01.014 PMC9989884 36659850

[33] Trinh A. L. Esposito A., “Biochemical resolving power of fluorescence lifetime imaging: untangling the roles of the instrument response function and photon-statistics,” Biomed. Opt. Express 12(7), 3775–3788 (2021). 10.1364/BOE.428070 PMC8367261 34457379

[34] Datta R. et al., “Fluorescence lifetime imaging microscopy: fundamentals and advances in instrumentation, analysis, and applications,” J. Biomed. Opt. 25(7), 071203 (2020). 10.1117/1.JBO.25.7.071203 PMC7219965 32406215

[35] Ahmed A. H. A. C. S. et al., “Fluorescence lifetime imaging microscopy by TCSPC (TD-FLIM),” in Practical Manual for Fluorescence Microscopy Techniques, PicoQuant (2016).

[r36] Gao D. et al., “FLIMJ: an open-source ImageJ toolkit for fluorescence lifetime image data analysis,” PLoS One 15(12), e0238327 (2020). POLNCL 1932-6203 10.1371/journal.pone.0238327 PMC7773231 33378370

[r37] Lazzari-Dean J. R. Miller E. W., “Optical estimation of absolute membrane potential using one- and two-photon fluorescence lifetime imaging microscopy,” Bioelectricity 3(3), 197–203 (2021). 10.1089/bioe.2021.0007 PMC8558063 34734167

[38] Tan K. K. D. et al., “Real-time open-source FLIM analysis,” Front. Bioinform. 3, 1286983 (2023). 10.3389/fbinf.2023.1286983 PMC10720713 38098814

[39] Link K. A. Penjweini R. Knutson J. R., “BPS2025—ultrafast fluorescence lifetimes of NADH via FLIM,” Biophys. J. 124(3), 330a (2025). BIOJAU 0006-3495 10.1016/j.bpj.2024.11.1817

[r40] Cao R. et al., “Optimization of FLIM imaging, fitting and analysis for auto-fluorescent NAD(P)H and FAD in cells and tissues,” Methods Appl. Fluoresc. 8(2), 024001 (2020). 10.1088/2050-6120/ab6f25 31972557

[41] Zhang J. et al., “Measuring metabolic changes in cancer cells using two-photon fluorescence lifetime imaging microscopy and machine-learning analysis,” J. Biophotonics 18(1), e202400426 (2025). 10.1002/jbio.202400426 PMC11700696 39587841

[42] Sharick J. T. et al., “Protein-bound NAD(P)H lifetime is sensitive to multiple fates of glucose carbon,” Sci. Rep. 8(1), 5456 (2018). 10.1038/s41598-018-23691-x PMC5883019 29615678

[43] Martinez J. et al., “FLIM quantification of temperature-dependent enzyme-NADH binding dynamics,” Biomed. Opt. Express 17(7), 3776–3791 (2026). BOEICL 2156-7085 10.1364/BOE.603469 PMC13372349 42460335

[44] Deng H. Zhadin N. Callender R., “Dynamics of protein ligand binding on multiple time scales: NADH binding to lactate dehydrogenase,” Biochemistry 40(13), 3767–3773 (2001). BICHAW 0006-2960 10.1021/bi0026268 11300756

[45] Torikata T. et al., “Lifetimes and NADH quenching of tryptophan fluorescence in pig heart lactate dehydrogenase,” Biochemistry 18(2), 385–390 (1979). BICHAW 0006-2960 10.1021/bi00569a024 217414

[46] Jones J. D. et al., “In vivo multiphoton microscopy detects longitudinal metabolic changes associated with delayed skin wound healing,” Commun. Biol. 1(1), 198 (2018). 10.1038/s42003-018-0206-4 PMC6242983 30480099

[47] Vargas-Ordaz E. et al., “Novel application of metabolic imaging of early embryos using a light-sheet on-a-chip device: a proof-of-concept study,” Hum. Reprod. 40(1), 41–55 (2025). 10.1093/humrep/deae249 PMC11700888 39521726

[r48] Nadiarnykh O. et al., “Carcinogenic damage to deoxyribonucleic acid is induced by near-infrared laser pulses in multiphoton microscopy via combination of two- and three-photon absorption,” J. Biomed. Opt. 17(11), 116024 (2012). 10.1117/1.JBO.17.11.116024 23214185

[49] Theodossiou A. Martinez J. Walsh A. J., “Fast autofluorescence imaging to evaluate dynamic changes in cell metabolism,” J. Biomed. Opt. 29(12), 126501 (2024). 10.1117/1.JBO.29.12.126501 PMC11657876 39703201

[50] Szulczewski J. M. et al., “In vivo visualization of stromal macrophages via label-free FLIM-based metabolite imaging,” Sci. Rep. 6(1), 25086 (2016). SRWSDA 10.1038/srep25086 PMC4879594 27220760

[51] Bi C. et al., “Stabilization of parameter estimates from multiexponential decay through extension into higher dimensions,” Sci. Rep. 12(1), 5773 (2022). 10.1038/s41598-022-08638-7 PMC8986819 35388008

